# Survival of Male Breast Cancer in Fars, South of Iran

**Published:** 2011-02-01

**Authors:** A Salehi, H Zeraati, K Mohammad, M Mahmoudi, A R Talei, A Ghaderi, M H Imanieh, A Fotouhi

**Affiliations:** 1 Department of Epidemiology and Biostatistics, School of Public Health, Tehran University of Medical Sciences, Tehran, Iran; 2 Department of Surgical Oncology, Shiraz University of Medical Sciences, Shiraz, Iran; 3 Department of Immunology, Shiraz Institute for Cancer Research, Shiraz University of Medical Sciences, Shiraz, Iran; 4 Gastroenterohepatology Research Center, Department of Pediatric Gastroenterology, Shiraz University of Medical Sciences, Shiraz, Iran

**Keywords:** Breast cancer, Male, Survival, Iran

## Abstract

**Background:**

Although breast cancer in men is uncommon, its incidence rate has an increasing trend. Due to its low incidence, there are few studies in this subject and limited information is available. The purpose of this study was to investigate clinicopathological characteristics and survival of male breast cancer (MBC) in Fars Province, south of Iran.

**Methods:**

The data for this study were obtained from the population based cancer registry of Vice-Chancellor for Health Affairs of Shiraz University of Medical Sciences and Shiraz hospitals between January 1, 1989 and January 1, 2008, including 64 patients with MBC. Demographic, clinical and pathological aspects were investigated. The Kaplan-Meier method was used for the determination of survival rate and Log Rank test for the comparison. The Cox proportional hazards model was used for the multiple analysis.

**Results:**

The patients’ mean age at the time of diagnosis was 60.3 years (SD=12.7). The most frequent age group (26.6%) was 51-60 years. The most common symptom (96.8%) was a palpable mass. The majority of patients (44.4%) had a symptom duration of less than or equal to 6 months. 56.3% of the patients had a tumor size of 2-4.9 cm. Forty six percent of the cases had axillary lymph node involvement. The median survival time was 10.0 years [95% confidence interval (CI): 6.0-14.0]. The 5 year overall survival rate was 66.0% (95% CI=51.0-81.0%). The median survival time of patients with axillary lymph node involvement was 8.2 years (95% CI=6.7-9.6) and for the cases without involvement was 12.0 years (95% CI=8.4-15.2). In addition to axillary lymph node involvement, positive family history in contrast to negative family history and left tumors in compari-son with right tumors were poorer prognostic factors in univariate analysis respectively (p=0.006, p=0.031). In multiple analysis, axillary lymph node involvement was an independent predictor of poorer survival (Hazard ratio=1.6, 95% CI=1.1-6.4, p=0.030) and the other variables did not have a significant effect.

**Conclusion:**

The mean age of MBC in this series is lower than that in western countries. It is compatible to the mean age of female breast cancer which is approximately one decade less than that in developed countries. The survival rate of MBC is relatively lower than that in western countries. Axillary lymph node involvement is an important prognostic factor in the survival of MBC. Multicenter population based studies with greater number of patients are required for better estimation of different aspects of MBC in Iran.

## introduction

According to Somerville (1952), the first reported case of male breast cancer (MBC) appeared in medical publication of Francisus Arcaeus (1493-1573).[1] Although breast cancer in men is uncommon and accounts for approximately 1% of all cases of malignancy in men, its incidence rate has had an increasing trend and has accelerated annually by 0.9% in the USA between 1975-2006, while the incidence rate of breast cancer in women has decreased by 2% per year between 1999- 2006.[2] The reasons for the increase are unknown and are not attributable to increased detection.[3] This increasing incidence is also present in other nations.[4] Death rate from MBC has remained essentially constant since 1975 while it has decreased in women by 1.9% annually between 1998-2006.[3] MBC cases had been 0.7% of all breast cancer cases and death of men due to breast cancer had been 1% of all breast cancer deaths in 2002 but these proportions have increased to 1.08% and 1.1% respectively in 2008.[4][5][6]

There is geographical variation in MBC occ rence. For example, MBC comprises 1% of all breast cancers in Europe and 0.4-0.6% in Korea.[3][7] In contrast, 6% of all breast cancer diagnoses in men in Tanzania and even higher percentages are reported in some other countries in central Africa.[8][9] MBC accounts for 0.65% of all cases of malignancy in men in Iran. There are 6674 incident cases of breast cancer diagnosed in Iran in 2007 of whom 3.26% were men. [10] In a hospital-based cancer registry in Fars Province, southern Iran, the crude incidence rate and ASR for MBC were reported 0.22 and 0.34 respectively. These figures for females were 11.58 and 18.06 respectively.[11]

Significant differences in the biological and clinicopathological characteristics have been described between male and female breast cancer.[4][12] Because of its low incidence, MBC has not been studied as extensively as female breast cancer. Appropriate management guidelines for MBC have not yet been clearly established, and limited information is available concerning the epidemiology, treatmen and prognosis of this disease. Therefore, the treatment guidelines have been extrapolated from the data based on female breast cancer.[13][14] It follows that the study of MBC becomes more important every day. In this study, we investigated clinicopathological characteristics and survival of MBC in Fars, southern Iran.

## Materials and Methods

The data for this study were obtained from the population based cancer registry of Vice-Chancellor for Health Affairs of Shiraz University of Medical Sciences and Shiraz hospitals between January 1, 1989 and January 1, 2008, including 64 patients with MBC. Their medical records were reviewed carefully. The data bank of Pathology Department was used to complete the information. The variables studied were demographic data including the patient age at diagnosis, job, marital status, and residence, clinical data including history of benign tumor, history of tobacco and alcohol use, type and duration of symptoms, and pathological data including tumor size, histological type, axillary lymph node involvement, laterality, chest wall invasion, staging and grading. The survival data were obtained from Death Registry of ViceChancellor for Health Affairs of Shiraz University of Medical Sciences and telephone contacts were made to complete the information. The tumor stage was based on the 6th American Joint Committee on ancer (AJCC) criteria. The grading followed Nottingham modification of the Bloom- Richardson System. SPSS (Chicago, IL, USA, Version 15) was used for statistical analysis. We used Kaplan-Meier method to determine the survival rate and Log Rank test for comparison. The Cox proportional hazards model was used for the multiple analysis. Right censor was applied from the final day to those who survived the study period, and other certain deaths for those who were lost to follow up. P value less than 5% was considered significant.

## Results

Patients' mean age at time of diagnosis was 60.3 years (12.7). Minimum and maximum ages of cases were 32 and 87 years. Forty five patients (70.0%) were diagnosed after the age of 50 years. The most frequent age group (26.6%) was 51-60 years. General characteristics of the patients are presented in Table 1.

The most common symptom was a palpable mass (96.8%). Ten patients (15.6%) had nipple discharge. Two cases had bilateral breast cancer. The majority of patients (44.4%) had a symptom duration of less than or equal to 6 months. The clinical characteristics of the patients are summarized in Table 2. Twenty seven patients (56.3%) had a tumor size of 2-4.9 cm. Forty six percent of the cases had axillary lymph node involvement. The pathologic characteristics are presented in Table 3.

Survival data for 56 patients (87%) were available. There were 19 deaths reported among the patients and 37 men were alive until the end of the study. Median follow-up time, from first pathological diagnosis until the time of death or the end of study, was 60 months. The median survival time was 10 years (95%CI: 6-14). The 3, 5 and 10 year overall survival rates were 83% (95% CI=72-94%), 66% (95% CI=51-81%) and 45% (95% CI=24-66%), respectively. In univariate analysis; axillary lymph node involvement, family history of breast cancer and laterality were predictor variables with significant effect on survival rate. The median survival time of patients with axillary lymph node involvement was 8.2 years (95% CI=6.7-9.6) and for the cases without involvement was 12.0 years (95% CI=8.4-15.2, p=0.007). Patients with positive family history had poorer survival (p=0.006). Survival rates of patients with right breast cancer were better than the survival rates of those with cancer on the left side (p=0.031). The median survival time according to the clinicopathological characteristics are summarized in Table 4. In multiple Cox regression, axillary lymph node involvement was an independent predictor of poorer survival (Hazard ratio=1.6, 95% CI=1.1-6.4) and the other variables had not significant effect.

**Table 1:** General characteristics of male breast cancer patients

**Characteristics**	**Patients (n=64)**	**%**
Age at Diagnosis (year)	64^a^	100
<40	5	7.8
41-50	14	21.9
51-60	17	26.6
61-70	13	20.3
71-80	12	18.7
>80	3	4.7
Residence	60	94.0
Fars	50	83.0
Other Provinces	10	17.0
Marital status	43	67.0
Married	42	98.0
Single	1	2.0
History of Benign tumor	61	95.0
Negative	60	98.0
Positive	1	2.0
History of Alcohol Use	58	90.0
Negative	42	72.4
Positive	16	27.6
History of Tobacco Use	42	65.6
No tobacco use	20	47.6
Current tobacco use	16	38.1
Past tobacco use	6	14.3

^a^ Total number available of the 64 subjects

**Table 2:** Clinical characteristics of male breast cancer patients

**Characteristics**	**Patients (n=64)**	**%**
Mass	64	100.0
Yes	62	96.8
No	2	3.2
Nipple Discharge	64	100.0
Yes	10	15.6
No	54	84.4
Nipple Ulceration	64	100.0
Yes	8	12.5
No	56	87.5
Nipple Retraction	64	100.0
Yes	7	11.0
No	57	89.0
Skin Fixation	64	100.0
Yes	10	15.7
No	54	84.3
Skin Ulceration	64	100.0
Yes	4	6.3
No	60	93.7
Skin Redness	64	100.0
Yes	7	11.0
No	57	89.0
Palpable Axillary Lymph Node	64	100.0
Yes	4	6.0
No	60	94.0
Arm Swelling	64	100.0
Yes	0	0.0
No	64	100.0
Duration of Symptoms (month)	45	70.3
Symptom≤6	20	44.4
6< Symptom ≤12	14	31.2
12< Symptom ≤24	6	13.3
24< Symptom	5	11.1

**Table 3:** Pathologic characteristics of male breast cancer patients

**Characteristics**	**Patients (n=64)**	**%**
Tumor Size	48	75.0
<2	9	18.7
2-4.9	27	56.3
>4.9	12	25.0
Stage	53	82.8
0	1	1.9
I	5	9.4
II	38	71.7
III	6	11.4
IV	3	5.6
Laterality	64	100.0
Left	31	48.4
Right	31	48.4
Bilateral	2	3.2
Chest wall Invasion	55	85.9
Negative	49	89.1
Positive	6	10. 9
Axillary LymphNode Involvement	59	92.2
Negative	32	54. 2
Positive	27	45.8
Histological Grade	46	71.8
I	19	41.3
II, III	27	58.7

**Table 4:** Survival of male breast cancer according to clinicopothological characteristics

**Characteristics**	**Number**	**Median Survival Time (years)**	**Log- rank test P-value**
Age (years)			0.670
≤50	16	9.6	
>50	38	11.2	
Family history			0.006
Negative	51	11.6	
Positive	2	7.3	
Alcohol Use			0.120
Negative	38	13.4	
Positive	15	8.4	
Tobacco Use			0.520
Negative	15	10.5	
Positive	19	7.2	
Symptom duration (months)			0.190
<6	15	12.5	
6-12	10	6.6	
>12	11	5.7	
Laterality			0.031
Left	28	8.4	
Right	25	13.5	
Tumor size (cm)			0.770
<2	7	10.1	
2-5	23	9.4	
>5	12	8.9	
Stage			0.490
I	6	10.0	
II	31	9.4	
III	7	9.1	
Grading			0.150
I	15	9.5	
II, III	24	7.6	
Axillary lymph node involvement			0.007
Negative	30	12.0	
Positive	27	8.2	

## Discussion

This study has shown that patients' mean age and survival rate of MBC in Iran is lower than western countries. The mean age at time of diagnosis in breast cancer is reported to be approximately 63 years in women and 68 years in men in North America and Western Europe.[15][16][17] According to two studies in Turkey, the mean age of MBC has been found to be 58 and 60 years.[18][19] In Japan and Korea, 62.5 and 58 years are reported for the mean age of MBC.[8][20] The mean age for the cases in our study was 60.3 years which is lower than that in western countries and similar to Asian study reports. It matches the mean age of female breast cancer in Iran which is approximately one decade less than that in developed countries.[21] As with women, the most common symptom of breast cancer is painless lump, which alone or with other problems arises in 50-97% of cases.[22][23] In this study, 96.8% of patients had breast lump.

Since male breast tissue is rudimentary, it does not usually differentiate a undergo lobular formation unless exposed to increased concentrations of endogenous or exogenous estrogen. Thus the predominant histological type of disease is invasive ductal, which makes up more than 90% of all male breast tumors.[15][24] Yoney et al. have reported 94.9% invasive ductal carcinoma in their study.[18] The histological type of all tumors of the patients in our study was invasive ductal carcinoma. In large reported series,most of the cases (54-58%) were grade II.[15] In our study, 54.3% of the patients were grade II.

The treatment strategy for breast cancer in men is the same as that in women and the primary treatment is mastectomy with axillary dissection.[25][26][27] In the past, radical mastectomy was preferred and the rationale for this was the localized lesion being close to the pectoralis major muscle and the tumor in a more advanced stage in men compared to that in women at the time of diagnosis.[28] Recent studies are in favor of modified radical or simple mastectomy combined with radiation therapy.[15][29] There seems to be no prominent difference in survival between these methods.[28] In our study, modified radical mastectomy was done for 55 cases (85.9%), and radical mastectomy for 5 patients (7.8%). Information on operation done on four of the patients was not available. Some studies suggested that breast cancer has a worse prognosis in men than in women, but if agematched and stage- matched breast cancer are compared, there is no difference between the sexes.[30] Five and 10 years overall survival of breast cancer in men is reported as 63-85.5% and 41-76% in different studies.[8][18][31] In Fars Province Hospital-based Cancer Registry, five-year overall survival was 58% (95% CI=53%–62%) in females.[32] In our study, 5 and 10 years overall survival rates were 66% and 45%, respectively. Different results have been reported regarding tumor size and survival of MBC. It has been argued that the large size of tumors can be considered a poor prognostic factor for surviv in MBC.[15] A fewstudies, of course, have found no relationship between tumor size and the prognosis.[18] In our study, there was no significant correlation between tumor size and survival. This could be due to the small number of patients or the effect of other prognostic factors.

The axillary lymph node involvement in malebreast cancer is reported approximately 55% in various studies and is considered a poor prognostic factor in most of them.[20][29][30] In our study, 46% of the patients had axillary lymph node involvement and their 5 years survival rate was 45% which is significantly lower than the 68% survival rate in cases without lymph node involvement.

To our knowledge, this is the first study to evaluate survival of MBC in Iran and it may be a basis for further necessary studies regarding this malignancy in our country. Limitations include retrospective design of the study, the lack of enough information regarding estrogen and progesterone receptors, inadequate number of patients and lack of detailed information due to radiotherapy and chemotherapy.

In conclusion, the mean age of MBC in this series is lower than that in western countries. It is compatible to the mean age of female breast cancer which is approximately one decade less than that in developed countries. The survival rate of MBC is relatively lower than that in western countries. There are few studies on MBC due to its rarity and information about different aspects of the disease is not enough, especially in the developing countries and further investigations are required, especially during multicenter studies with a greater number of patients.
